# Elucidating the Mechanism of the Liqi Yangyin Formula in Treating Depression–Constipation Comorbidity: An Integrative Approach Using Network Pharmacology and Experimental Validation

**DOI:** 10.3390/ph19010106

**Published:** 2026-01-07

**Authors:** Lianjie Xu, Shun Seng Ong, Xiaoyue Deng, Yunzhi Qian, Zhao Tang, Ming Li, Tianshu Xu

**Affiliations:** 1 Department of Traditional Chinese Medicine, Nanjing Drum Tower Hospital, The Drum Tower Clinical Medical College, Nanjing University of Chinese Medicine, Nanjing 210008, China; 2 Department of Nutrition, University of North Carolina at Chapel Hill, Chapel Hill, NC 27599, USA; 3 Department of Traditional Chinese Medicine, Nanjing Drum Tower Hospital, Affiliated Hospital of Medical School, Nanjing University, Nanjing 210008, China

**Keywords:** Liqi Yangyin formula, depression, constipation, network pharmacology, JAK2/STAT3 pathway, neuronal apoptosis, inflammation

## Abstract

**Background**: The traditional formula Liqi Yangyin (LQYY) has shown clinical and preclinical efficacy for depression with constipation, yet its molecular mechanisms remain incompletely defined. This study aimed to elucidate its mechanisms using an integrative approach. **Methods**: Constituents of LQYY were profiled by UPLC-MS/MS and integrated with network pharmacology and molecular docking to identify brain-accessible components and putative targets. A chronic unpredictable mild stress (CUMS) model was used for experimental validation. Outcomes included behavioral tests (sucrose preference test, open field test, and forced swimming test), gastrointestinal indices, including fecal water content, time of first black stool, and intestinal propulsion rate, histopathology of the prefrontal cortex (PFC) and colon, TUNEL staining, NeuN immunofluorescence, Western blotting, and qRT-PCR. **Results**: LQYY attenuated CUMS-induced weight loss and depressive-like behaviors and improved intestinal transit metrics. It reduced neuronal apoptosis in the PFC and ameliorated colonic injury. Mechanistically, docking and enrichment analyses highlighted hub targets (STAT3, AKT1, ESR1, IL-6, TNF, TP53) and the JAK/STAT pathway. In vivo, LQYY decreased IL-6, TNF-α, ESR1, TP53, and STAT3, and increased AKT1 in the PFC and colon; it also reduced the TUNEL-positive rate and restored NeuN labeling, upregulated Bcl-2, and downregulated p-JAK2/JAK2 and p-STAT3/STAT3 ratios, and the expression of Bax and cleaved-caspase-3 in the PFC, consistent with the suppression of pro-inflammatory and apoptotic signaling. **Conclusions**: LQYY exerts antidepressant and pro-motility effects in CUMS mice by modulating JAK2/STAT3-centered networks and inhibiting neuronal apoptosis, thus supporting a multi-component, multi-target strategy for treating depression with constipation, and providing a defined molecular hypothesis for future investigation.

## 1. Introduction

Depression is a chronic, debilitating mood disorder characterized by persistent low mood, cognitive dysfunction, anhedonia, and disturbances of sleep and appetite; in severe cases, suicidal ideation may occur [1]. Prolonged depressive illness exerts substantial effects on both the digestive and nervous systems. Constipation—one of the most common gastrointestinal disorders—is defined by reduced bowel frequency, straining, incomplete evacuation, or the passage of dry, hard stools [2]. In clinical practice, depression and constipation frequently co-occur, and a considerable proportion of patients experience both conditions simultaneously [3]. Moreover, the severity of constipation correlates with the intensity of depressive symptoms, collectively diminishing quality of life and complicating management [4]. Although pharmacotherapies are available, they are limited by suboptimal remission rates, poor adherence, and notable adverse effects [5,6]. These gaps underscore the need to clarify the pathogenesis of this comorbidity and to develop interventions that address both conditions concurrently.

Traditional Chinese medicine (TCM), with its multi-component, multi-target, and multi-pathway characteristics, offers a potentially advantageous approach with comparatively mild side effects. The Liqi Yangyin (LQYY) formula, composed of 12 herbs including *Adenophora triphylla* (Thunb.) A.DC. (Nanshashen, 20 g), *Ophiopogon japonicus* (Thunb.) Ker Gawl. (Maidong, 20 g), *Scrophularia ningpoensis* Hemsl. (Xuanshen, 30 g), *Rehmannia glutinosa* (Gaertn.) Libosch. ex DC. (Shudihuang, 30 g), *Prunus armeniaca* L. (Xingren, 10 g), *Atractylodes macrocephala* Koidz. (Baizhu, 40 g), *Citrus × aurantium* L. (Zhiqiao, 15 g), *Magnolia officinalis* Rehder & E.H.Wilson (Houpo, 10 g), *Trichosanthes kirilowii* Maxim. (Gualouren, 20 g), *Cannabis sativa* L. (Huomaren, 10 g), *Prunus humilis* Bunge (Yuliren, 15 g), and *Dolomiaea costus* (Falc.) Kasana & A.K.Pandey (Muxiang, 8 g) (https://mpns.kew.org)—traditionally used to regulate qi, alleviate depressed mood, nourish yin, and promote defecation—has shown clinical utility for constipation [7]. Mechanistic evidence suggests that LQYY can rebalance the gut microbiota, increase short-chain fatty acid (SCFA) levels, stimulate enterochromaffin cells (ECs) to release serotonin (5-HT), strengthen the intestinal barrier, enhance intestinal motility, and thereby relieve constipation [8,9]. Furthermore, bioactive constituents of LQYY, such as naringin and hesperidin, display antidepressant activity in preclinical models [10,11,12,13]. Moreover, the TCM principle of “treating different diseases with the same method,” together with the modern concept of the brain–gut axis, provides a theoretical rationale for applying LQYY to depression with comorbid constipation. However, evidence directly addressing this comorbidity remains limited, hindering broader clinical adoption.

To interrogate complex herbal mechanisms, network pharmacology—integrating systems biology with computational analysis—has emerged as a useful strategy for constructing drug component–gene–disease interaction networks and identifying key signaling nodes [14,15]. Nonetheless, target predictions derived solely from network pharmacology are not fully reliable [16], necessitating corroboration by molecular docking and experimental validation.

In this study, we sought to elucidate how LQYY exerts therapeutic effects in depression with constipation. We first profiled LQYY by UPLC-MS/MS and screened for blood–brain barrier (BBB)-permeable constituents using TCMSP and SwissADME. We then applied network pharmacology to predict candidate targets and pathways, followed by molecular docking to assess interactions between core constituents and core targets. Finally, we validated these predictions in a chronic unpredictable mild stress (CUMS) mouse model, delineating potential targets and pathways through which LQYY may ameliorate depressive and constipatory phenotypes.

## 2. Results

### 2.1. UPLC-MS/MS Profiling of LQYY Compounds

**Figure 1:**
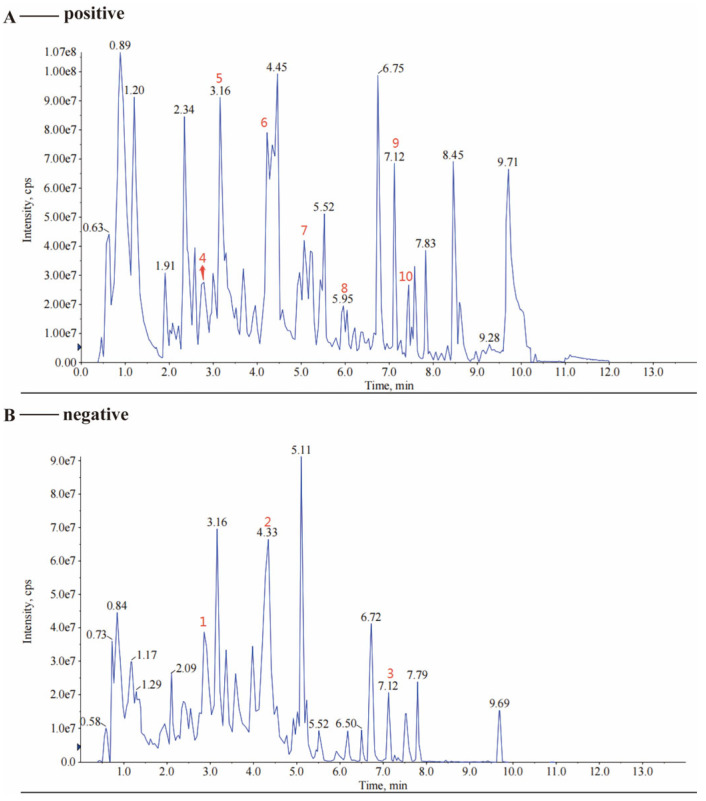
Representative total ion chromatograms (TICs) of the LQYY extract acquired by UPLC-MS/MS. (**A**) Positive ion mode. (**B**) Negative ion mode. Key identified compounds are annotated: 1. Coniferin (*Citrus × aurantium* L.). 2. Umbelliferone (*Adenophora triphylla* (Thunb.) A.DC., *Citrus × aurantium* L.). 3. Embelin (unassigned). 4. Tryptamine (multi-herb origin). 5. Stepharine (*Prunus armeniaca* L.). 6. 6-Hydroxycoumarin (*Adenophora triphylla* (Thunb.) A.DC., *Citrus × aurantium* L.). 7. Coumarin (*Citrus × aurantium* L.). 8. Hesperetin (*Citrus × aurantium* L.). 9. Nobiletin (*Citrus × aurantium* L.). 10. Elemicin (*Dolomiaea costus* (Falc.) Kasana & A.K.Pandey).

Samples were analyzed on UPLC-MS/MS platforms (AB SCIEX TripleTOF 6600 for qualitative profiling and AB SCIEX 4500 QTRAP for quantitative MRM, SCIEX, Framingham, MA, USA), with metabolite annotation referenced to the MetWare database. This workflow enabled sensitive detection and confident classification of constituents. As shown in Figure 1, the detected compounds encompassed multiple classes, predominantly terpenoids, alkaloids, flavonoids, and phenolic acids.

### 2.2. Screening of LQYY Active Ingredients and Prediction of Related Targets

After deduplication, 116 candidate bioactive constituents predicted to be BBB-permeable were retained—25 from TCMSP and 91 from SwissADME. Putative targets for these constituents (754 unique genes) were compiled from TCMSP, SwissTargetPrediction, and SymMap (Supplementary Material S1).

### 2.3. Identification of Disease Targets for Depression with Constipation

**Figure 2:**
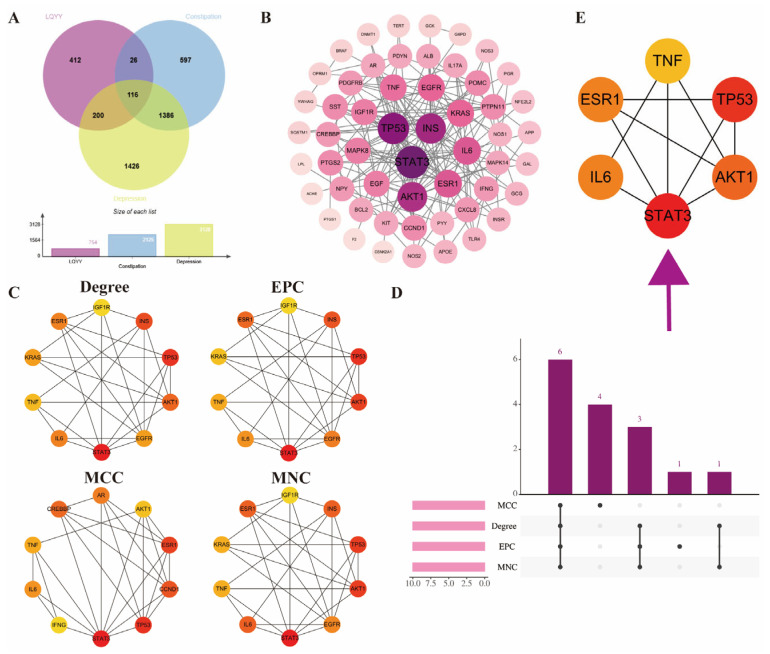
PPI analysis of LQYY targets in depression with constipation and hub gene identification. (**A**) Venn diagram showing the overlap between disease-associated targets and targets of BBB-permeable LQYY constituents. (**B**) STRING-derived PPI network visualized in Cytoscape. (**C**) Top 10 ranked nodes by Degree, EPC, MCC, and MNC (CytoHubba). (**D**,**E**) UpSet plot highlighting six consensus hub genes—STAT3, AKT1, ESR1, IL-6, TNF, and TP53—identified by all four algorithms.

After deduplication, 3128 depression-associated targets and 2125 constipation-associated targets were retrieved from TTD, GeneCards, and DisGeNET (Supplementary Material S2). Intersecting these disease targets with the predicted targets of BBB-permeable LQYY constituents yielded 116 overlapping targets (Figure 2A), which were used for subsequent network construction and enrichment analyses.

### 2.4. PPI Network Construction and Hub Gene Identification

The overlapping targets were submitted to STRING to construct a PPI network, which was imported into Cytoscape for visualization and analysis (Figure 2B). Hub genes were ranked in CytoHubba using four metrics—Degree, EPC, MCC, and MNC—and the top 10 from each list were retained (Figure 2C). An UpSet analysis identified six consensus hub genes shared by all four algorithms: STAT3, AKT1, ESR1, IL-6, TNF, and TP53 (Figure 2D,E).

### 2.5. GO and KEGG Pathway Enrichment Analyses

**Figure 3:**
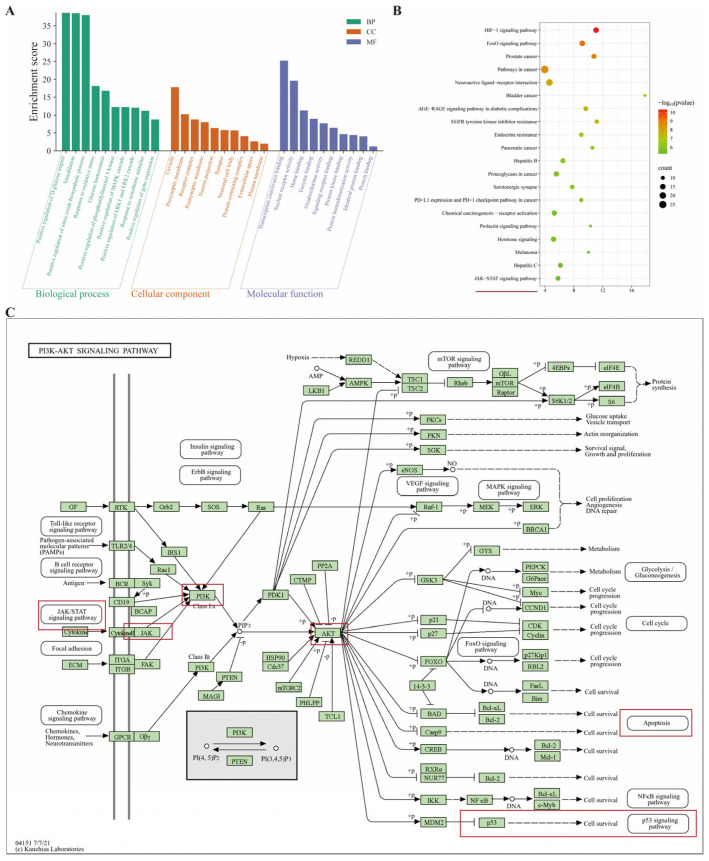
GO and KEGG enrichment analyses of the 116 intersecting targets. (**A**) Top 10 significantly enriched GO terms across the three categories—BP, CC, and MF. (**B**) KEGG bubble plot of the top 20 enriched pathways. JAK-STAT signaling pathways (highlighted in red) were selected for further investigation. (**C**) Schematic highlighting the PI3K-AKT pathway as a bridge linking hub genes to the JAK-STAT cascade.

GO (*q* < 0.05) and KEGG (*p* < 0.05) enrichment analyses were performed on the 116 intersecting targets using the DAVID database and visualized with the Bioinformatics platform. The top 10 terms for each GO category are shown in Figure 3A. In Biological Process (BP), the top enriched terms included response to oxidative stress, positive regulation of MAPK cascade, positive regulation of ERK1/ERK2 cascade, and response to xenobiotic stimulus. In Cellular Component (CC), targets localized predominantly to the presynaptic membrane, receptor complex, postsynaptic membrane, and neuron projection. In Molecular Function (MF), enriched terms included transcription coactivator binding, nuclear receptor activity, signaling receptor binding, and protein homodimerization activity.

KEGG analysis identified the top 20 pathways (Figure 3B). Among these, HIF-1, FoxO, and JAK-STAT signaling pathways are closely linked to inflammatory responses and apoptosis. Notably, pathways related to neurotransmission and neuronal excitability were also enriched, including neuroactive ligand–receptor interaction and serotonergic synapse. Integrating these findings with the PPI results, we infer that LQYY may alleviate symptoms of depression with constipation by modulating inflammatory signaling, apoptosis, and neurotransmitter pathways. Although the PI3K-AKT pathway itself did not rank within the top 20, our network analysis revealed that it functionally bridges several core hub genes (e.g., AKT1, TP53) with the JAK-STAT cascade (Figure 3C); this suggested a potential crosstalk mechanism. However, given the stronger enrichment signal and more direct literature support for the JAK-STAT pathway in inflammation and apoptosis, we prioritized it for subsequent experimental validation.

### 2.6. Component–Target–Pathway–Disease Network

**Figure 4:**
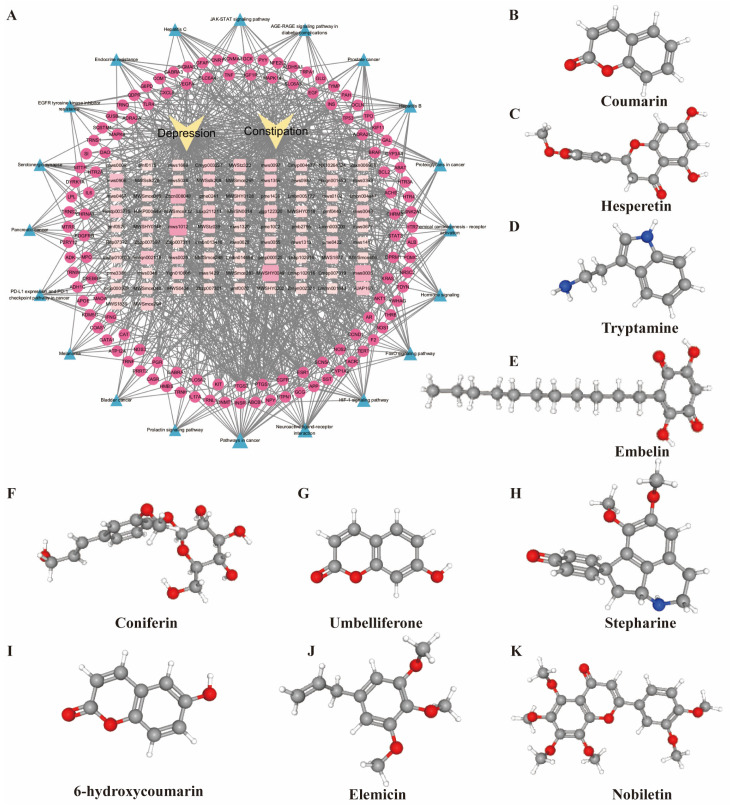
Component–target–pathway–disease network and structures of key LQYY constituents. (**A**) Integrated network for depression with constipation constructed in Cytoscape. Rectangles denote BBB-permeable LQYY components; circles denote disease-associated targets (depression and constipation); triangles denote KEGG pathways. Node size and color reflect degree centrality (larger/darker = higher connectivity). (**B**) Coumarin (mws1012). (**C**) Hesperetin (MWSHY0049). (**D**) Tryptamine (mws0005). (**E**) Embelin (Zbzn008048). (**F**) Coniferin (mws0906). (**G**) Umbelliferone (Hmqn002118). (**H**) Stepharine (HJAP166). (**I**) 6-Hydroxycoumarin (zjgp122320). (**J**) Elemicin (MWSmce294). (**K**) Nobiletin (mws0043). The above 3D structures are all from the pubchem (https://pubchem.ncbi.nlm.nih.gov/ (accessed on 20 August 2025)) database.

An integrated component–target–pathway–disease network was constructed in Cytoscape (Figure 4A), visualizing putative relationships among 83 BBB-permeable constituents, 116 candidate targets, and enriched pathways. Network topology (degree centrality) highlighted several core constituents—most notably coumarin, hesperetin, and nobiletin—derived primarily from *Magnolia officinalis* Rehder & E.H.Wilson and *Citrus × aurantium* L. (Figure 4B–K). High-degree compounds tended to connect with a larger fraction of targets, suggesting that they may represent key pharmacodynamic drivers of LQYY’s effects in depression with constipation. Most targets in this network overlapped with the PPI results and are closely associated with inflammatory signaling and apoptosis.

### 2.7. Molecular Docking

**Figure 5:**
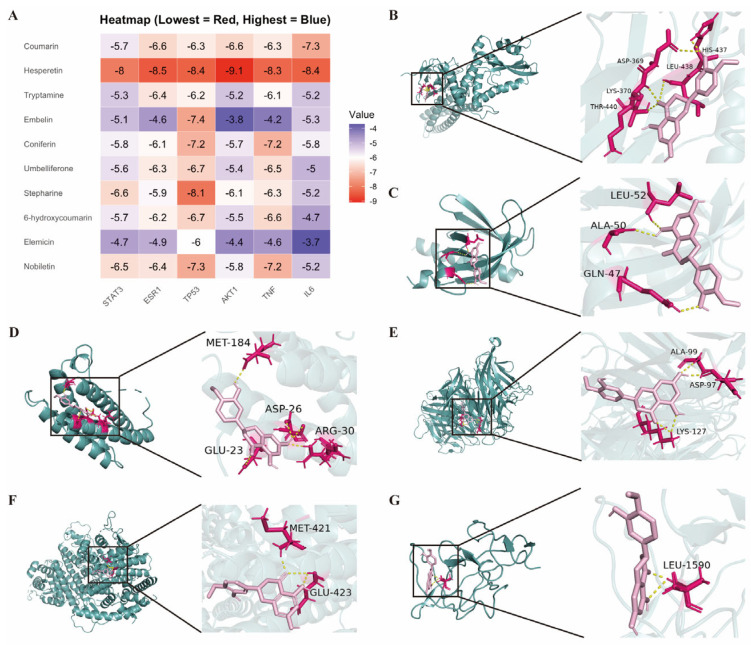
Molecular docking of key LQYY constituents with hub targets. (**A**) Heatmap of AutoDock binding energies (kcal·mol^−1^); more negative values indicate stronger predicted affinity. (**B**) Hesperetin–STAT3. (**C**) Hesperetin–AKT1. (**D**) Hesperetin–IL-6. (**E**) Hesperetin–TNF. (**F**) Hesperetin–ESR1. (**G**) Hesperetin–TP53. The 3D poses illustrate representative ligand–target interactions.

Molecular docking was performed between the top 10 candidate constituents and the six consensus hub targets, yielding docking energies for each ligand–target pair (Figure 5A). In this scoring scheme (AutoDock), more negative values indicate stronger predicted binding and greater complex stability. Six compounds—coumarin, tryptamine, coniferin, hesperetin, stepharine, and nobiletin—showed binding energies < −5.0 kcal·mol^−1^ with the core targets. Among them, hesperetin exhibited the most favorable overall profile; representative 3D docking poses for hesperetin with each target are shown in Figure 5B-G. Including AKT1, hesperetin engaged Leu52, Ala50, and Gln47, forming a compact hydrogen-bond network within the ligand-binding cavity. ESR1 showed hydrophobic and polar contacts involving Met421 and Glu423, while TP53 displayed a key interaction with Leu1590. These residue-level interactions corroborate the highly negative docking energies (−8.0 to −9.1 kcal/mol) and support hesperetin’s potential to directly modulate inflammatory and apoptotic signaling proteins. Collectively, the docking results reinforce the role of hesperetin as a major bioactive constituent contributing to the multi-target therapeutic effects of LQYY.

### 2.8. LQYY Attenuates CUMS-Induced Depression-Like Behaviors and Pathological Damage of PFC in Mice

**Figure 6:**
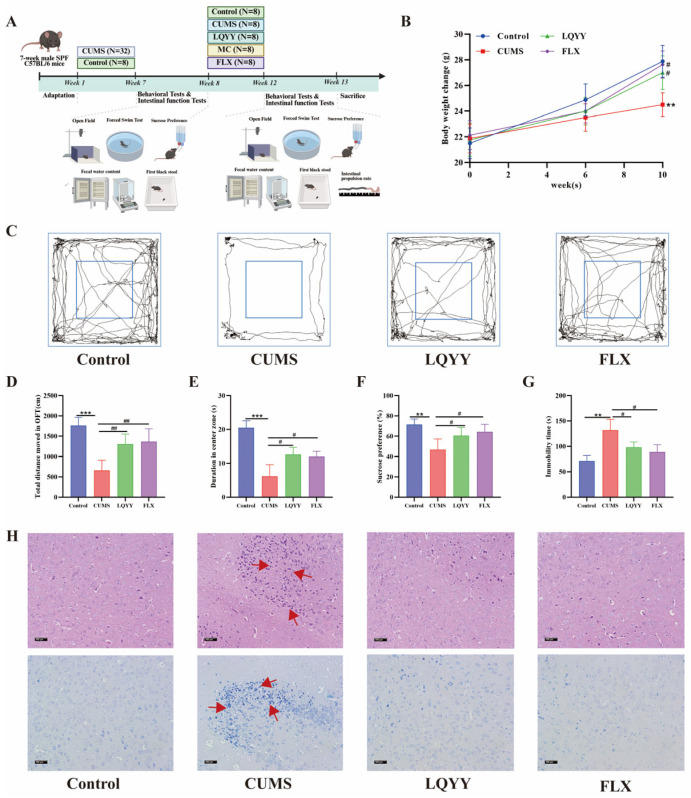
LQYY ameliorates CUMS-induced depression-like behaviors and neuronal pathology. (**A**) Experimental timeline. (**B**) Body weight across the study (*n* = 8). (**C**) Representative OFT trajectories. (**D**) OFT total distance (*n* = 8). (**E**) OFT time in center zone (*n* = 8). (**F**) SPT (*n* = 8). (**G**) FST immobility time (*n* = 8). (**H**) H&E and Nissl staining of PFC sections (×200; scale bar, 100 μm); arrows indicate changes in cell morphology, with the cells showing shrinkage and nuclear fragmentation (*n* = 3). ** *p* < 0.01 and *** *p* < 0.001 vs. Control; ^#^ *p* < 0.05 and ^##^ *p* < 0.01 vs. CUMS.

The CUMS model was used to evaluate LQYY after 6 weeks of stress exposure (Figure 6A). Compared with Controls, CUMS mice showed typical depression-like phenotypes: reduced body weight, lower sucrose preference, decreased total distance traveled and time in the center zone in the OFT (with thigmotactic trajectories), and increased immobility in the FST. Treatment with LQYY or FLX significantly mitigated these abnormalities (Figure 6B–G). Consistently, mice subjected to CUMS exhibited significant neuronal pathology in the PFC, characterized by disorganized neuronal soma arrangement, nuclear pyknosis, and deep nuclear staining, and Nissl staining revealed a marked reduction or loss of Nissl bodies in PFC neurons, whereas LQYY and FLX attenuated these histopathological changes (Figure 6H). These histopathological improvements provide direct visual evidence of LQYY’s neuroprotective efficacy, effectively bridging our molecular findings on apoptosis inhibition with the recovery of behavioral functions.

### 2.9. LQYY Alleviates CUMS-Induced Constipation and Colonic Pathological Damage in Depression Mice

**Figure 7:**
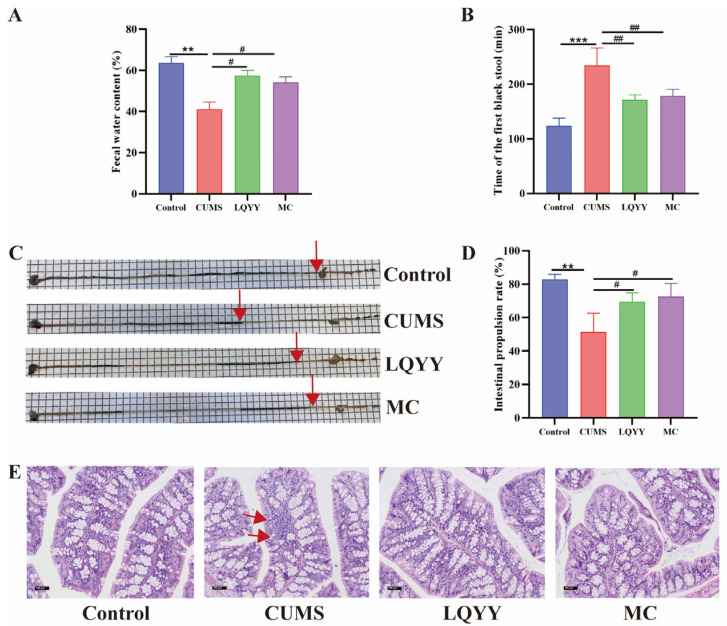
LQYY ameliorates CUMS-induced impairment of intestinal transit and colonic injury in mice. (**A**) Fecal water content (*n* = 8). (**B**) Time to first black stool (*n* = 8). (**C**) Representative images of charcoal progression; red arrows mark the charcoal front. (**D**) Intestinal propulsion rate (*n* = 8). (**E**) H&E-stained colonic sections (×200; scale bar, 100 μm), with arrows indicating places of injury (*n* = 3). ** *p* < 0.01 and *** *p* < 0.001 vs. Control; ^#^ *p* < 0.05 and ^##^ *p* < 0.01 vs. CUMS.

After behavioral testing, gastrointestinal transit was evaluated. Compared with Controls, CUMS markedly reduced fecal water content and intestinal propulsion rate and prolonged the time to first black stool; treatment with LQYY or MC significantly reversed these changes (Figure 7A–D). Histologically, CUMS induced colonic mucosal damage—epithelial disruption, lamina propria injury, and inflammatory cell infiltration—whereas LQYY and MC attenuated inflammatory infiltration and structural injury (Figure 7E). These findings indicate that LQYY mitigates CUMS-induced constipation and improves colonic pathology in this depression model.

### 2.10. Effects of LQYY on JAK2/STAT3 Signaling and Related Targets

**Figure 8:**
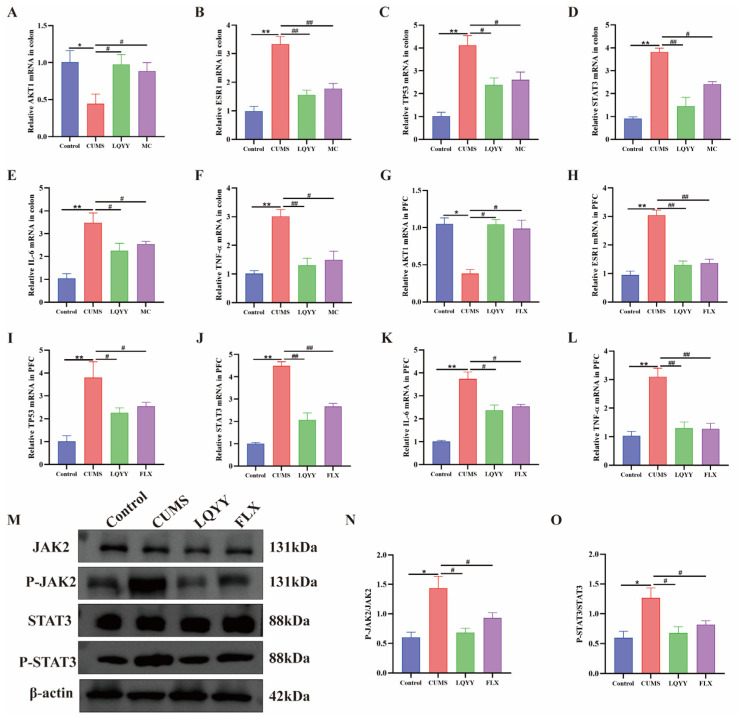
LQYY modulates JAK2/STAT3 signaling and related targets in CUMS-induced mice. (**A**–**F**) Relative mRNA expression of AKT1, ESR1, TP53, STAT3, IL-6, and TNF-α in colon (*n* = 6). (**G**–**L**) Relative mRNA expression of AKT1, ESR1, TP53, STAT3, IL-6, and TNF-α in PFC (*n* = 6). (**M**–**O**) Western blot analysis of JAK2, p-JAK2, STAT3, and p-STAT3 in PFC (*n* = 3). * *p* < 0.05 and ** *p* < 0.01 vs. Control; ^#^ *p* < 0.05 and ^##^ *p* < 0.01 vs. CUMS.

KEGG enrichment highlighted the JAK-STAT pathway as a key axis potentially mediating the effects of LQYY in depression with constipation. Consistently, molecular docking supported high-affinity interactions between core LQYY constituents and hub proteins, including AKT1, STAT3, IL-6, TNF, ESR1, and TP53. Guided by these findings, we quantified AKT1, IL-6, TNF-α, ESR1, TP53, and STAT3 mRNA in the colon and PFC. qRT-PCR showed that CUMS increased IL-6, TNF-α, ESR1, TP53, and STAT3 transcripts and decreased AKT1 relative to Controls; LQYY and FLX (or MC) reversed these changes (Figure 8A–L). At the protein level, Western blotting demonstrated that LQYY and FLX significantly reduced phospho-JAK2/JAK2 and phospho-STAT3/STAT3 ratios versus the CUMS model (Figure 8M–O). Together with the enrichment and docking data, these results indicate that LQYY ameliorates behavioral and gastrointestinal phenotypes by down-modulating JAK2/STAT3 signaling and regulating ESR1, IL-6, TNF-α, AKT1, STAT3, and TP53.

### 2.11. LQYY Attenuates PFC Neuronal Apoptosis in CUMS Mice

**Figure 9:**
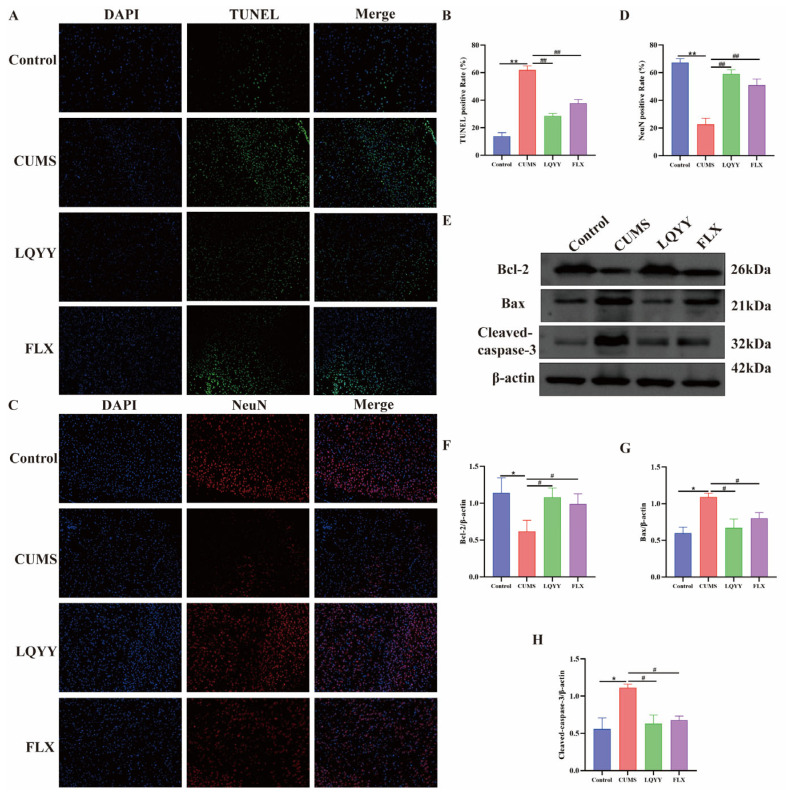
LQYY inhibits neuronal apoptosis in the PFC. (**A**) Representative TUNEL staining of PFC (20×; scale bar, 50 μm; *n* = 3). (**B**) Quantification of TUNEL-positive rate. (**C**) Immunofluorescence of NeuN in PFC (20×; scale bar, 50 μm; *n* = 3). (**D**) Quantification of NeuN-positive rate. (**E**–**H**) Western blots of Bcl-2, Bax, and cleaved caspase-3 in PFC (*n* = 3). * *p* < 0.05 and ** *p* < 0.01 vs. Control; ^#^ *p* < 0.05 and ^##^ *p* < 0.01 vs. CUMS.

Apoptosis contributes to neuronal injury and depression-like behaviors. Consistent with H&E findings of PFC pathology in CUMS mice, TUNEL staining and NeuN immunofluorescence revealed increased TUNEL-positive cells and reduced NeuN-labeled neurons after CUMS. Treatment with LQYY or FLX significantly reduced TUNEL positivity and restored NeuN labeling (Figure 9A–D). To further assess the relevant indicators of apoptosis, we examined the expression of Bcl-2, Bax, and cleaved caspase-3 in PFC. CUMS elevated the pro-apoptotic markers Bax and cleaved caspase-3 while reducing the anti-apoptotic protein Bcl-2. LQYY and FLX reversed these changes, indicating attenuation of neuronal apoptosis (Figure 9E–H).

## 3. Discussion

Clinically, constipation in patients with depression is often attributable to antidepressant use; a meta-analysis reported a significantly higher incidence of constipation among antidepressant users than placebo recipients [5]. In addition, depressive symptoms are frequently accompanied by reduced appetite and altered dietary habits, leading to inadequate intake of dietary fiber and fluids and thereby increasing constipation risk [17]. Animal studies further demonstrate that depressive models exhibit disordered intestinal motility [18]. Indeed, CUMS-induced depression in mice produces a constellation of constipation-related phenotypes, including reduced intestinal transit and fecal water content [19]. Although conventional pharmacotherapies can address individual symptoms, their overall effectiveness in this complex comorbidity remains limited by modest efficacy, adverse effects, and the lack of integrated treatments that improve both mood and bowel function. Consequently, there is growing interest in complementary approaches—particularly TCM—to meet this unmet need [20]. In this study, we combined UPLC-MS/MS profiling, network pharmacology, and molecular docking to identify candidate bioactive constituents of LQYY and to predict their disease-relevant targets and pathways, followed by in vivo validation.

Our analyses highlighted several brain-accessible constituents—coumarin, hesperetin, coniferin, stepharine, 6-hydroxycoumarin, and nobiletin—as potential mediators of LQYY’s effects in depression with constipation. As summarized in Supplementary Table S1, these core components belong to diverse chemical classes and their known activities (e.g., anti-inflammatory, neuroprotective, pro-motility) collectively cover multiple pathological aspects of depression–constipation comorbidity, which strongly supports the multi-component, multi-target synergistic mechanism of LQYY from a chemical and pharmacological perspective. Prior work indicates that certain coumarins and derivatives possess favorable BBB permeability profiles, suggesting promising pharmacokinetics [21]. Coumarin exhibits broad pharmacological activities (antimicrobial, anti-inflammatory, anticonvulsant, anticancer, and monoamine oxidase inhibition) and can ameliorate depressive-like behavior by elevating monoaminergic neurotransmission and suppressing pro-inflammatory cytokine synthesis via inhibition of MAPK signaling [22,23]. Hesperetin, a citrus flavonoid, exerts anti-inflammatory, antioxidant, anti-apoptotic, and neuroprotective effects and improves depressive symptoms and memory impairment in preclinical models [24,25]. Relatedly, hesperidin enhances colonic motility in loperamide-induced constipation by activating the 5-HT_4_ receptor and increasing intracellular Ca^2+^, thereby upregulating cAMP/PKA and p-CREB signaling [26]. Nobiletin, a polymethoxyflavonoid, crosses the BBB and confers neuroprotection in various models [27]; it also reduces MAPT expression in colon tissue, lowers TNF-α and IL-6 levels, protects interstitial cells of Cajal (ICCs) from apoptosis to relieve constipation, and mitigates depressive-like behaviors [28]. Collectively, these findings support the concept that LQYY acts through multiple constituents, targets, and pathways to modulate both central and enteric processes relevant to depression with constipation.

Stress exposure in rodents and humans is associated with synaptic loss, neuronal atrophy, and glial alterations in the PFC [29]. The PFC—accounting for ~30% of the entire cerebral cortex—governs higher cognitive and affective functions, including decision-making, emotion regulation, and social behavior [30,31]. Neuroimaging consistently shows reduced perfusion and metabolism in the PFC of individuals with depression [32], implicating this region as a key node for translating stress into affective and cognitive dysfunction [33]. Under chronic stress, PFC microglia adopt a pro-inflammatory phenotype and release cytokines such as IL-6 and TNF-α, disrupting neuron–glia homeostasis and exacerbating depressive-like behaviors [34]. Moreover, gut-derived metabolites (e.g., SCFAs), neurotransmitters, and immunomodulators can traverse the BBB and directly influence neuronal function and plasticity in the PFC [35]. For these reasons, we selected the PFC as the primary brain region for assessing depression-related endpoints.

Network pharmacology and PPI analyses identified STAT3, TP53, ESR1, AKT1, TNF, and IL-6 as hub targets—largely linked to inflammatory signaling and apoptosis. ESR1, a nuclear receptor mediating estrogen signaling, regulates physiological and pathological processes across reproductive, cardiovascular, endocrine, nervous, and immune systems and is implicated in multiple endocrine disorders and cancers [36,37]. Estrogen acting via ESR1 modulates depressive-like behaviors by influencing neurotransmitter turnover and 5-HT receptor function [38]; ESR1 expression is also elevated in constipation-predominant irritable bowel syndrome, implicating it in bowel dysfunction [39]. AKT1, a key effector of the PI3K/AKT pathway, phosphorylates diverse substrates to regulate neuronal growth and survival and to restrain apoptosis [40]. PI3K/AKT signaling exerts antidepressant-like effects through downstream survival pathways; for example, ligustilide engages AKT1, ESR1, and PI3K/AKT to produce antidepressant effects [41]. In the gut, PI3K/AKT restores enteric neuronal integrity and motility, and pterostilbene alleviates loperamide-induced constipation by protecting ICCs via PI3K/AKT activation [42,43]. TP53 encodes the tumor suppressor p53, which orchestrates DNA repair, metabolism, senescence, and apoptosis—partly by increasing the Bax/Bcl-2 ratio [44,45]. Genetic variation at TP53 (e.g., 72C allele) may influence depression risk by modulating cell survival and apoptosis [46], and TP53 downregulation in the colon has been linked to therapeutic benefits in slow-transit constipation models [47]. Consistent with these lines of evidence, our docking indicated that many top LQYY constituents interact with ESR1, AKT1, and TP53, with hesperetin displaying the strongest predicted affinities (−8.5, −9.1, and −8.4 kcal·mol^−1^, respectively). Experimentally, ESR1 and TP53 mRNA were elevated and AKT1 reduced in the PFC and colon of CUMS mice; LQYY and FLX (or MC) reversed these changes, supporting the involvement of these targets in LQYY’s actions.

Apoptosis is a central contributor to stress-related neuronal injury. KEGG enrichment in our study indicated that LQYY may act on the JAK/STAT pathway. This pathway comprises tyrosine kinase-associated receptors, JAKs (JAK1/2/3, TYK2), and STATs (STAT1/2/3/4/5A/5B/6), with JAK2/STAT3 being extensively studied in cellular responses to extracellular cues [48]. Under stress, microglia become activated and release pro-inflammatory cytokines, notably IL-6 and TNF-α [49]. At this time, the gut microbiota is disrupted, and gut microbiota imbalance may contribute to intestinal inflammation [50].

IL-6 and TNF-α levels are elevated in depression, and their receptor complexes signal via JAK/STAT: receptor-associated JAK2 is phosphorylated (p-JAK2), which in turn phosphorylates STAT3 (p-STAT3), driving transcriptional programs that regulate inflammation, autophagy, and apoptosis—processes that collectively influence neuronal integrity [51,52]. In line with this mechanism, CUMS increased expression of IL-6, TNF-α, and STAT3 in the PFC and colon, and elevated p-JAK2/JAK2 and p-STAT3/STAT3 ratios in the PFC, whereas LQYY and FLX (or MC) reduced these indices. At the cellular level, apoptosis reflects an imbalance between pro-apoptotic Bax and anti-apoptotic Bcl-2; when this balance shifts toward Bax, caspase cascades are engaged [53]. Cleaved caspase-3—the activated form—is a canonical executioner and widely used marker of apoptosis [54]. Consistently, LQYY inhibited neuronal apoptosis, as conclusively demonstrated by the multi-level evidence in Figure 9. The TUNEL staining and NeuN immunofluorescence offer direct evidence of reduced neuronal death and preserved neuronal integrity. In parallel, the Western blot analysis of Bax, Bcl-2, and cleaved caspase-3 confirmed the underlying molecular mechanism, showing that LQYY restores the pro-/anti-apoptotic balance and inhibits the final execution phase of apoptosis. This convergence of evidence solidifies the conclusion that the suppression of neuronal apoptosis is a critical endpoint of JAK2/STAT3 pathway inhibition by LQYY, ultimately contributing to the amelioration of depressive-like behaviors.

This study acknowledges three primary limitations. Firstly, the quantification of key bioactive components hinged on relative UPLC-MS/MS data due to a lack of pure standards, necessitating future work to determine absolute concentrations for standardization and advanced pharmacokinetic and pharmacodynamic studies. Secondly, and directly related, the absence of systematic toxicological and pharmacokinetic data for LQYY must be addressed. Subsequent research should therefore prioritize in vivo safety evaluation and characterization of the absorption, distribution, metabolism, and excretion of its core active constituents to robustly support clinical translation. Finally, our integrative approach was optimized to pinpoint the most prominent bioactive components and core pathways, such as hesperetin and the JAK-STAT axis. However, it does not allow us to definitively rule out the contribution of any single ingredient within the LQYY formula. The synergistic nature of TCM means that herbs not highlighted by our network pharmacology screening may play crucial supporting roles, such as enhancing the bioavailability of active compounds or modulating ancillary targets. Future studies employing more reductionist designs would be required to fully deconvolute the contribution of each constituent.

Building on these analyses, a pivotal conclusion can be drawn. Our integrative data predict that the therapeutic efficacy of the LQYY formula is likely attributable to a focused subset of its constituents. Specifically, the network pharmacology and molecular docking identified coumarin, tryptamine, coniferin, hesperetin, stepharine, and nobiletin as core bioactive compounds, which are derived from *Prunus armeniaca* L. (Xingren), *Dolomiaea costus* (Falc.) Kasana & A.K.Pandey (Muxiang), *Citrus × aurantium* L. (Zhiqiao), and *Adenophora triphylla* (Thunb.) A.DC. (Nanshashen), as confirmed by our UPLC-MS/MS profiling (Figure 1). Consequently, a definitive future study is planned to directly test this hypothesis by preparing a fixed combination of these six compounds at doses equivalent to their quantified content in the LQYY extract, followed by a parallel experimental assessment of its efficacy against the whole extract in the CUMS model.

Despite these limitations, our integrative approach supports a multi-target mechanism whereby LQYY modulates inflammatory and apoptotic signaling and influences neurotransmission, yielding concurrent improvements in mood-related behaviors and gastrointestinal transit. These findings provide a mechanistic rationale for further translational evaluation of LQYY in depression with constipation.

## 4. Materials and Methods

### 4.1. Rationale, Preparation, and UPLC-MS/MS Analysis of LQYY

The LQYY formula is designed based on the TCM pathogenesis of depression–constipation comorbidity, which is primarily identified as “Qi Stagnation with Intestinal Dryness” (Qiyu Changzao). The corresponding therapeutic principle is to “Regulate Qi and Nourish Yin” (Liqi Yangyin), where *Adenophora triphylla* (Thunb.) A.DC. (Nanshashen), *Ophiopogon japonicus* (Thunb.) Ker Gawl. (Maidong), and *Scrophularia ningpoensis* Hemsl. (Xuanshen) serve to replenish fluids, supported by *Cannabis sativa* L. (Huomaren) and *Prunus humilis* Bunge (Yuliren), to directly lubricate the bowels. *Prunus armeniaca* L. (Xingren) and *Trichosanthes kirilowii* Maxim. (Gualouren) work to restore the lung’s descending function that motivates the intestines, aided by *Atractylodes macrocephala* Koidz. (Baizhu), used to strengthen the spleen’s function in transporting fluids, and Libosch. ex DC. (Shudihuang), utilized to nourish the kidney’s yin, consolidating the foundation for fluid production. *Dolomiaea costus* (Falc.) Kasana & A.K.Pandey (Muxiang), *Magnolia officinalis* Rehder & E.H.Wilson (Houpo), and *Citrus × aurantium* L. (Zhiqiao) act to resolve the qi stagnation.

The crude materials for the LQYY formula were purchased from Jiangsu Huahong Pharmaceutical Technology Co., Ltd. (Zhenjiang, China), and marked with their respective batch numbers: Nanshashen (Cat: 240620); Maidong (Cat: 241203); Xuanshen (Cat: 240905); Shudihuang (Cat: 241012); Baizhu (Cat: 250331); Zhiqiao (Cat: 241111); Houpo (Cat: 250205); Gualouren (Cat: 250217); Huomaren (Cat: 241209); Yuliren (Cat: 241218); Muxiang (Cat: 230710); and Xingren (Cat: 241115). The total crude drug weight (228 g) was soaked in water for 45 min and decocted twice, and the combined filtrates were concentrated to a final concentration of 3 g/mL.

For metabolite extraction, 200 µL of the filtrate was transferred to a 1.5 mL microcentrifuge tube and mixed with 200 µL of 70% methanol containing the internal standard. Samples were vortex-mixed for 15 min at 12,000 rpm at 4 °C and centrifuged for 3 min. The supernatant was subjected to UPLC-ESI-MS/MS. Chromatographic analysis was performed on a UPLC system coupled via electrospray ionization to a triple quadrupole/linear ion trap (QTRAP) mass spectrometer. Separation used an Agilent SB-C18 column (2.1 mm × 100 mm, 1.8 µm). Mobile phase A was water with 0.1% formic acid; mobile phase B was acetonitrile with 0.1% formic acid. The gradient program was as follows: 0.0 min, 95:5 (A:B); ramp to 5:95 by 12.0 min; and re-equilibrate to 95:5 by 14.0 min. Flow rate was 0.35 mL·min^−1^; injection volume was 2 µL. ESI source settings were as follows: temperature, 500 °C; ion spray voltage, +5500 V (positive mode) and -4500 V (negative mode); and source gas 1, source gas 2, and curtain gas at 50, 60, and 25 psi, respectively. Multiple reaction monitoring (MRM) transitions were acquired with compound-specific declustering potentials (DPs) and collision energies (CEs) optimized for each ion pair.

### 4.2. Network Pharmacology

#### 4.2.1. Screening of LQYY Bioactive Compounds and Target Prediction

To ensure comprehensive and accurate coverage, all compounds detected by UPLC-MS/MS were screened in the Traditional Chinese Medicine Systems Pharmacology database (TCMSP; https://tcmsp-e.com/tcmsp.php (accessed on 5 June 2025)). Retention criteria were oral bioavailability (OB) ≥ 30%, drug-likeness (DL) ≥ 0.18, and BBB permeability ≥ −0.30. For independent pharmacokinetic and drug-likeness assessment, SMILES strings of the detected compounds were retrieved from PubChem and evaluated in SwissADME (http://www.swissadme.ch/ (accessed on 5 June 2025)). Compounds were retained if gastrointestinal (GI) absorption was classified as “High” and BBB permeation as “Yes.” For drug-likeness, compounds meeting at least two of the following rules were kept: Lipinski, Ghose, Veber, Egan, and Muegge. Putative targets of the retained compounds were then predicted using SwissTargetPrediction (http://www.swisstargetprediction.ch/ (accessed on 5 June 2025)), and only targets with a predicted probability > 0.12 were included for further analysis. Compound–target information was additionally cross-referenced in SymMap (http://www.symmap.org/search/ (accessed on 5 June 2025)). All target identifiers were standardized using the UniProt database (https://www.uniprot.org (accessed on 5 June 2025)) to ensure consistent protein nomenclature.

#### 4.2.2. Prediction of Disease-Related Targets for Depression with Constipation

To compile disease-associated genes relevant to depression with constipation, we queried three public resources—GeneCards (https://www.genecards.org/ (accessed on 6 June 2025)), DisGeNET (https://www.disgenet.org/ (accessed on 6 June 2025)), and the Therapeutic Target Database (TTD; http://db.idrblab.net/ttd/ (accessed on 6 June 2025))—using the keywords “depressive disorder” and “constipation.” Target lists from the three databases were merged and deduplicated to generate a nonredundant, high-confidence dataset for subsequent analyses.

#### 4.2.3. Construction of the Protein–Protein Interaction (PPI) Network

The intersecting targets of LQYY and depression with constipation were submitted to STRING (https://cn.string-db.org/ (accessed on 6 June 2025)) to construct a PPI network. The organism was set to *Homo sapiens*, and the minimum required interaction score was defined as high confidence (>0.900). The resulting network was exported and visualized in Cytoscape (v3.10.3).

#### 4.2.4. Identification of Hub Genes

After constructing the PPI network, node centrality was evaluated in CytoHubba (Cytoscape) using four algorithms: Degree, Edge Percolated Component (EPC), Maximal Clique Centrality (MCC), and Maximum Neighborhood Component (MNC). For each metric, the top 10 ranked genes were retained. The four lists were then integrated by a consensus approach (overlap of top-ranked nodes) to define the final set of hub genes.

#### 4.2.5. Enrichment Analysis of GO and KEGG

To characterize the functions of the 116 intersecting genes between LQYY and depression with constipation, we submitted the gene list to the DAVID Bioinformatics Resources for Gene Ontology (GO) enrichment and Kyoto Encyclopedia of Genes and Genomes (KEGG) pathway analyses. These analyses were used to infer relevant biological processes and signaling pathways. Results were visualized with an online bioinformatics platform (https://www.bioinformatics.com.cn/ (accessed on 7 June 2025)). GO terms were summarized in a bar chart across the three categories—Biological Process (BP), Cellular Component (CC), and Molecular Function (MF)—and KEGG pathway enrichment was presented as a bubble plot.

#### 4.2.6. Construction of the Component–Target–Pathway–Disease Network

LQYY candidate components, putative targets, disease-related targets (depression and constipation), and the top 20 enriched signaling pathways were imported into Cytoscape (v3.10.3) to construct an integrated component–target–pathway–disease network. Nodes denoted components, targets, pathways, and diseases (distinguished by shape/color), and edges represented documented or predicted associations between node pairs. This visualization provides an intuitive overview of multi-component, multi-target interactions and pathway convergence.

### 4.3. Molecular Docking

Molecular docking was used to validate compound–target interactions and estimate binding affinities. The six consensus hub proteins identified by CytoHubba were treated as receptor targets, and the ten highest-degree components from the component–target–pathway–disease network were selected as key ligands. Three-dimensional ligand structures were obtained from PubChem, and protein crystal structures were retrieved from the Protein Data Bank (PDB). Protein structures were prepared in PyMOL (v3.0.3) by removing crystallographic water and nonessential ions and by adding hydrogens. Docking was performed with AutoDock (v1.5.7), and the best-scoring pose for each ligand–target pair was retained. Docking poses and interactions were visualized in PyMOL.

### 4.4. Experimental Validation

#### 4.4.1. Animals and Interventions

Seven-week-old, specific-pathogen-free (SPF) male C57BL/6 mice were purchased from Jiangsu Huachuang Xinnuo Pharmaceutical Science and Technology Co., Ltd., Taizhou, China. Animals were housed under controlled conditions (23 ± 2 °C; ~60% relative humidity; 12 h light/12 h dark cycle). All procedures complied with institutional guidelines and were approved by the Laboratory Animal Ethics Committee of Nanjing Drum Tower Hospital, Nanjing University Medical School (No. 2024AE01019).

A CUMS model was used as previously described [24]. Mice were exposed daily, for six weeks, to a random pair of two distinct stressors selected from the following: 24 h water and food deprivation; 45° cage tilt (24 h); empty-cage exposure (24 h); horizontal shaking (10 min); swimming in 40 °C water (10 min); reversal of the light/dark cycle (24 h); tail pinch (5 min); wet bedding (24 h); and restraint (3 h).

After a 7-day acclimation, mice were randomized into five groups (*n* = 8/group): Control, CUMS, LQYY, mosapride citrate (MC), and fluoxetine (FLX). Except for the Control group, all groups underwent the 6-week CUMS procedure and then received daily oral treatment for 4 weeks: LQYY (30 g·kg^−1^·day^−1^), MC (3 mg·kg^−1^·day^−1^; H19990313; Chengdu Kanghong Pharmaceutical Group Co., Ltd., Chengdu, China), or FLX (10 mg·kg^−1^·day^−1^; HJ20181215; Patheon France, Évreux, France). The dose of LQYY was determined by converting the clinical human dose to a mouse-equivalent dose based on body surface area, followed by confirmation in a pilot dose-finding experiment, and the doses of MC and FLX were selected based on studies and verified in our laboratory [55]. The treatment period was set according to previous studies, which is sufficient to observe neuronal apoptosis and treatment effects [54,56]. Body weight was recorded weekly. Following treatment, behavioral assessments were performed, including the sucrose preference test (SPT), open field test (OFT), and forced swim test (FST). Gastrointestinal outcomes included the time of first black stool and measurement of fecal water content. Mice were then euthanized by cervical dislocation. Segments of colon and prefrontal cortex (PFC) were fixed in 4% paraformaldehyde for histology; the remaining PFC and colonic tissues were snap-frozen and stored at −80 °C for subsequent analyses.

#### 4.4.2. Sucrose Preference Test (SPT)

The SPT was used to assess anhedonia. The procedure comprised habituation and testing phases. For habituation, mice were first given two bottles containing 1% (*w*/*v*) sucrose for 24 h, followed by 24 h with one bottle of 1% sucrose and one bottle of water; bottle positions were alternated randomly. After 24 h of food and water deprivation, the formal test was conducted by providing one bottle of 1% sucrose and one bottle of water, with positions exchanged every 6 h. Over a 12 h period, consumption of sucrose (C1) and water (C2) was recorded for each mouse. Sucrose preference (%) was calculated as follows: sucrose preference = C1/(C1 + C2) × 100.

#### 4.4.3. Open Field Test (OFT)

The OFT was used to evaluate anxiety- and locomotion-related behaviors. Mice were placed in a 45 cm × 45 cm × 45 cm arena for a 5 min acclimation, and then returned to the starting position for a 5 min recording session. Movements were tracked with an automated system (TopScan v3.0, Clever Sys Inc., Reston, VA, USA). Outcomes included time spent in the central zone and total distance traveled. The apparatus was cleaned with 75% ethanol between trials to eliminate olfactory cues.

#### 4.4.4. Forced Swimming Test (FST)

The FST was performed as described previously [33]. Each mouse was placed individually in a transparent cylinder (height 25 cm, diameter 10 cm) containing 20 cm of water maintained at 25 ± 1 °C. Behavior was recorded for 6 min in a quiet room. Post-test, the immobility time of each mouse during the final 4 min of swimming was measured using a stopwatch. Immobility is defined as the period during which mice cease active struggling and only make slight limb movements necessary to keep their heads above water.

#### 4.4.5. Fecal Water Content

Fresh fecal pellets were collected into pre-weighed 2 mL tubes. Wet weight (W1) was determined as the weight of tube + wet feces minus the empty tube weight. Samples were dried at 100 °C for 3 h in a thermostatic oven, and dry weight (W2) was recorded as tube + dried feces minus the empty tube weight. Fecal water content (%) was calculated as follows: FWC = (W1 − W2)/W1 × 100.

#### 4.4.6. First Black Stool

A charcoal solution was prepared by dissolving gum acacia (1 g) in 80 mL distilled water with heating until clear, adding kaolin (5 g), boiling the mixture three times, cooling to room temperature, and adjusting to 100 mL with distilled water to obtain a stable suspending vehicle. An activated carbon (charcoal) suspension (e.g., 5% *w*/*v*) was prepared with this vehicle and mixed thoroughly before use. After a 24 h fast (water ad libitum), mice received the charcoal solution by oral gavage at 0.1 mL per 10 g body weight. Animals were then housed individually for observation. The administration time (T1) and time of first black fecal pellet (T2) were recorded. Time (min) = T2 − T1.

#### 4.4.7. Intestinal Propulsion Rate

To assess intestinal transit, mice were fasted for 12 h with free access to water and then gavaged with a 5% (*w*/*v*) activated carbon suspension at 0.1 mL per 10 g body weight. Thirty minutes later, mice were euthanized by cervical dislocation. The gastrointestinal tract was excised en bloc from the pylorus to the anus. Total intestinal length (L1) and the migration distance of the charcoal front (L2) were measured. The intestinal propulsion rate (%) was calculated as follows: IPR = L2/L1 × 100.

#### 4.4.8. Histopathological Testing of PFC and Colon

PFC and colonic tissues were fixed in 4% paraformaldehyde for 48 h, embedded in paraffin, and sectioned at 4 µm. PFC and colonic tissue sections were stained via hematoxylin–eosin (H&E) to evaluate histological structure. Additionally, PFC tissue sections underwent Nissl staining to assess neuronal structure and damage. Slides were scanned on a Zeiss MDS bright-field scanning microscope, and histopathological changes in the PFC and colon were evaluated.

#### 4.4.9. Quantitative Real-Time PCR

Total RNA was isolated from PFC and colonic tissues using Trizol reagent (15596026CN, Invitrogen) and reverse-transcribed into cDNA using the ReverTra Ace qPCR RT kit (FSQ-301, ToYoBo). Quantitative PCR (qPCR) was performed on a QuantStudio™ 5 Real-Time PCR System using SYBR Green Realtime PCR Master Mix (QPK-201, ToYoBo) and gene-specific primers for mouse IL-6, TNF-α, ESR1, AKT1, TP53, STAT3, and β-actin. Primer sequences (5′ to 3′) were as follows: IL-6—forward TCCATCCAGTTGCCTTCTTG, reverse AAGCCTCCGACTTGTGAAGTG; TNF-α—forward ACTGGCAGAAGAGGCACTCC, reverse GCCACAAGCAGGAATGAGAA; ESR1—forward CCGCCTTCTACAGGTCTAAT, reverse CACAGTAGCGAGTCTCCTTG; AKT1—forward CTGCCCTTCTACAACCAGGA, reverse CATACACATCCTGCCACACG; TP53—forward ACTGCATGGACGATCTGTTG, reverse GCCATAGTTGCCCTGGTAAG; STAT3—forward CCGGCCCTTAGTCATCAAGA, reverse CTTTTGTGTTCGTGCCCAGA; and β-actin—forward CACGATGGAGGGGCCGGACTCATC, reverse TAAAGACCTCTATGCCAACACAGT. Relative mRNA expression was calculated by the 2^−ΔΔCt^ method using β-actin as the endogenous control. Primers were synthesized by Tsingke Biotech Co., Ltd. (Beijing, China).

#### 4.4.10. TUNEL Staining

Paraffin-embedded PFC sections were deparaffinized in xylene, rehydrated through graded ethanol to water, and treated with Proteinase K. Sections were rinsed with PBS and gently air-dried. TUNEL reaction mixture (G1510, Servicebio, Wuhan, China) was applied per the manufacturer’s instructions and incubated at 37 °C in a humidified chamber. After incubation, sections were washed with PBS, counterstained with DAPI, mounted with antifade medium, and imaged using a THUNDER imaging system (Leica Microsystems, Wetzlar, Germany).

#### 4.4.11. Immunofluorescence Staining

Paraffin sections (4 µm) of PFC were deparaffinized in xylene, rehydrated with ethanol and water, and subjected to heat-induced antigen retrieval in citrate buffer (pH 6.0). After PBS washes, endogenous peroxidase activity was quenched with 3% H_2_O_2_ for 15 min at room temperature, followed by additional PBS washes. Sections were incubated with primary anti-NeuN antibody (381075, Zenbio), and then with an appropriate fluorescent secondary antibody. Nuclei were counterstained with DAPI, slides were mounted with antifade medium, and images were acquired on a THUNDER imaging system (Leica Microsystems, Wetzlar, Germany).

#### 4.4.12. Western Blot Analysis

PFC tissue was lysed in RIPA buffer (Servicebio, Wuhan, China), and protein concentration was determined using a BCA assay (Servicebio, Wuhan, China). The proteins were resolved by 10% SDS-PAGE (PAGE Color Gel Ultra Rapid Preparation Kit, Servicebio, Wuhan, China) and transferred to PVDF membranes. Membranes were blocked with 5% skim milk for 2 h at room temperature and incubated overnight at 4 °C with primary antibodies against JAK2 (R24775, Zenbio), phospho-JAK2 (R381556, Zenbio), STAT3 (R22785, Zenbio), phospho-STAT3 (R381552, Zenbio), Bax (AF0120, Affinity), Bcl-2 (AF6139, Affinity), and cleaved caspase-3 (341034, Zenbio). After three washes with TBST, membranes were incubated with the appropriate secondary antibodies for 1 h at room temperature. Signals were detected using enhanced chemiluminescence (ECL), and band intensities were quantified with ImageJ (version 1.51j8; Java 1.8.0_112, 64-bit).

#### 4.4.13. Statistical Analysis

Analyses were performed using IBM SPSS Statistics 25.0 and GraphPad Prism 9.5. Data are expressed as mean ± SD from at least three independent experiments. Group differences were assessed by one-way ANOVA followed by Tukey’s multiple-comparisons test. When normality or homoscedasticity assumptions were violated, the Kruskal–Wallis test (k independent samples) was applied. A *p* value < 0.05 was considered statistically significant.

## 5. Conclusions

This study integrated UPLC-MS/MS profiling, network pharmacology, molecular docking, and in vivo validation to elucidate how LQYY may act against depression with constipation. We identified several BBB-permeable constituents—coumarin, tryptamine, coniferin, hesperetin, stepharine, and nobiletin—and linked them to hub targets and signaling pathways. Convergent evidence highlighted JAK2/STAT3 as a key axis, with hesperetin showing the most favorable predicted binding across core proteins. Functionally, LQYY reversed CUMS-induced molecular abnormalities in the colon and PFC, reducing ESR1, TP53, IL-6, TNF-α, and STAT3, increasing AKT1, and lowering p-JAK2/JAK2 and p-STAT3/STAT3 ratios, alongside attenuating PFC neuronal apoptosis. Together, these findings support a multi-component, multi-target mechanism by which LQYY modulates inflammatory and apoptotic signaling to concurrently improve mood-related behaviors and gastrointestinal transit. The work provides mechanistic rationale and a translational foundation for the clinical exploration of LQYY in depression with constipation.

## Data Availability

The original contributions presented in this study are included in the article/Supplementary Materials. Further inquiries can be directed to the corresponding authors.

## Supplementary Materials

The following supporting information can be downloaded at https://www.mdpi.com/article/10.3390/ph19010106/s1: S1: Putative targets for bioactive constituents were compiled from TCMSP, SwissTargetPrediction, and SymMap. S2: Depression-associated targets and constipation-associated targets were retrieved from TTD, GeneCards, and DisGeNET. Table S1: Several of the most effective classes of compounds and their pharmacological activities.
