# Supplementary material for: Elucidating the Mechanism of the Liqi Yangyin Formula in Treating Depression–Constipation Comorbidity: An Integrative Approach Using Network Pharmacology and Experimental Validation
Source: Pharmaceuticals (Basel). 2026 Jan 7;19(1):106. doi: 10.3390/ph19010106 (PMC12844872; doi:10.3390/ph19010106)
Supplement: Supplementary file 1 [file pharmaceuticals-19-00106-s001.zip › Supplementary Table S1.pdf]

Supplementary Table 1: Several of the most effective classes of compounds and their pharmacological activities

Compound name	Classes of compound	Pharmacological activitys
Coumarin	Coumarins	favorable BBB permeability profiles;antimicrobial; anti-inflammatory; monoamine oxidase inhibition; anti-depressant
Hesperetin	Flavanones	anti-inflammatory; antioxidant; anti-apoptotic; neuroprotective effects; anti-depressant; improve constipation
Coniferin	Alcohol compounds	anti-inflammatory; oxidative stress-reducing; anti-apoptotic
Stepharine	Isoquinoline alkaloids	anti-neuroinflammatory; anti-aging; analgesic properties
Nobiletin	Flavones	crosses the BBB; confers neuroprotection; anti-depressant; improve constipation
Tryptamine	Plumerane	treatment-resistant depression; major depressive disorder; post-traumatic stress disorder
